# Assessing Outcomes After Adrenalectomy for Primary Aldosteronism – Early is Accurate: Retrospective Cohort Study

**DOI:** 10.1097/SLA.0000000000005639

**Published:** 2022-07-27

**Authors:** Diederik P.D. Suurd, Wessel M.C.M. Vorselaars, Dirk-Jan Van Beek, Inne H.M. Borel Rinkes, Wilko Spiering, Gerlof D. Valk, Menno R. Vriens

**Affiliations:** * Department of Surgical Oncology and Endocrine Surgery, University Medical Center Utrecht, Utrecht, the Netherlands; † Department of Vascular Medicine, University Medical Center Utrecht, Utrecht, the Netherlands; ‡ Department of Endocrine Oncology, University Medical Center Utrecht, Utrecht, the Netherlands

**Keywords:** adrenalectomy, antihypertensives, blood pressure, follow-up, hypertension, mixed model, primary aldosteronism

## Abstract

We aimed to investigate the postoperative trend in blood pressure (BP)-related outcomes [BP and antihypertensive (AHT) drug use] during the year following adrenalectomy for primary aldosteronism (PA) to determine the optimal timing for outcome assessment and to determine the necessary follow-up length.

**Background:**

Since the course of BP-related outcomes after adrenalectomy is unknown, the optimal timing of outcome assessment and follow-up duration are not clear.

**Methods:**

In this retrospective single center cohort study, we used a prospectively collected database with all patients referred for difficult-to-control-hypertension-analysis. All patients diagnosed with PA who underwent adrenalectomy were included. AHT drug use [in defined daily dose (DDD)] and home blood pressure measurements (HBPMs) during the first postoperative year were collected. A mixed-effects model was developed to assess the stability of DDD and HBPM over time and adjust for potential confounders.

**Results:**

In total 1784 patients were assessed for difficult-to-control-hypertension of whom 41 were included. Both the DDD and HBPM showed the strongest decrease in the first postoperative month (mean 1.6DDD; mean 140/85 mm Hg) compared with preoperative values (4.5DDD; 153/92 mm Hg). Thereafter, both outcomes showed a stable course from 4 to 6 months (1.6DDD; 136/86 mm Hg) up to 12 months postoperatively (2.0DDD; 136/83 mm Hg).

**Conclusions:**

This study showed that AHT drug use and HBPM decreased substantially within the first month after adrenalectomy for PA and afterwards generally remained stable during the year following adrenalectomy. We propose that BP-related outcomes can be assessed reliably early after adrenalectomy and question the need for routine long-term follow-up in referral centers.

Primary aldosteronism (PA) has an estimated prevalence of 3% to 13% among the hypertensive population.^1^,^2^ PA is preferably treated with adrenalectomy in case of a unilateral aldosterone-producing adenoma. The goals of adrenalectomy are to correct aldosteronism and hypertension. Although complete biochemical success is achieved in the majority of the patients, cure of hypertension is reported in only 27% to 37% of the patients.^3^–^6^ For clinicians working in the field of PA, the optimal timing to determine the effect of adrenalectomy on blood pressure (BP)-related outcomes [BP and antihypertensive drug (AHT) use] is uncertain. Consequently, the necessary duration of follow-up is not clear for patients and physicians.

Due to a lack of robust scientific evidence, the timing and duration of postoperative BP monitoring are guided by expert consensus. Nevertheless, the 2016 Endocrine Society Clinical Practice Guideline (CPG) suggests monitoring of BP 1 to 6 months after surgery.^7^ The Primary Aldosteronism Surgical Outcome (PASO) study group states that initial outcome assessment should be performed within 3 months and final outcome assessment between 6 and 12 months postoperatively.^5^ In a previous systematic review analyzing the trend of postoperative BP-related outcomes, the greatest drop in BP was observed in the first month(s) after surgery which seemed to stabilize thereafter.^8^ However, studies showed great variety in follow-up practices (eg, frequency of visits), the majority of studies were of limited methodological quality, and the use of AHT was generally not reported or underreported.

Therefore, we aimed to investigate the postoperative trend of BP-related outcomes in the first postoperative year after adrenalectomy for PA to determine the ideal timing for outcome assessment and to determine the necessary follow-up length. We hypothesized that BP-related outcomes after adrenalectomy for PA decrease early after surgery and will thereafter remain stable during the year following adrenalectomy in the majority of the patients.

## METHODS

This study is reported with guidance of the Strengthening The Reporting of OBservational Studies in Epidemiology (STROBE) statement.^9^

### Patient Selection

For this retrospective cohort study, the prospectively collected data including all consecutive patients with difficult-to-control hypertension referred to the Department of Vascular Medicine of the University Medical Center Utrecht, The Netherlands, between January 2010 and November 2020 was used. Difficult-to-control hypertension was defined as hypertension unresponsive to treatment according to the current guidelines and/or the presence of end-organ damage or vascular complications.^10^ All patients who underwent the “Analysis of Complicated Hypertension” diagnostic protocol were evaluated and all patients diagnosed with PA who underwent unilateral adrenalectomy in our center were included. Retrospectively, these patients were followed during the year following adrenalectomy. Approval for this study was given by Medical Ethics Committee of the University Medical Center Utrecht (The Netherlands). The need for formal informed consent was waived according to the Medical Research Involving Human Subjects Acts (Dutch Law: WMO, art. 1b).

### Analysis of Complicated Hypertension Diagnostic Protocol and Work-up of PA

The “Analysis of Complicated Hypertension” diagnostic protocol is a standardized protocol designed for the diagnosis of – or to rule out – secondary forms of hypertension including PA. The protocol has been extensively described before.^10^–^12^ The diagnosis of PA was in accordance with the Endocrine Society CPG recommendations.^7^ In brief, patients underwent extensive screening of medical history and lifestyle, office BP measurements (OBPM) on and without AHT, 24-h ambulatory BP measurement (ABPM) at home on and without AHT, home blood pressure measurements (HBPM) without AHT, biochemical laboratory measurements [including aldosterone-to-renin ratio (ARR)], vascular ultrasound screening, electrocardiogram, sleep apnea and therapy adherence questionnaires, and a saline infusion test (SIT). The SIT was performed regardless of ARR outcome. Plasma aldosterone concentration (pmol/L) and plasma renin activity (fmol/L/s) were measured after stopping interfering AHT. Details about the measurement methods are described in Supplemental Data 1, Supplemental Digital Content 1, http://links.lww.com/SLA/E108.

Computed tomography (CT) and/or adrenal venous sampling (AVS) were performed to distinguish between a unilateral aldosterone-producing adenoma (APA) or bilateral adrenal hyperplasia according to the Endocrine Society CPG. Patients underwent a posterior retroperitoneoscopic total adrenalectomy, however, when unfeasible an open total adrenalectomy was performed.

### Outcome Definitions

Follow-up data regarding AHT and HBPM were collected preoperatively and for at least 12 months after the adrenalectomy, stratified into 6 postoperative periods of follow-up (<1, 1–2, 2–4, 4–6, 6–12, and >12 mo). Frequency of follow-up visits were based on individual care plan. The type and dosage of AHT were used to calculate the “defined daily dose” (DDD), which is a measure of the average maintenance dose for a drug. Calculation of DDD was based on the WHO ATC/DDD index 2021 (https://www.whocc.no/atc_ddd_index). HBPM were measured within daily practice care and not via standardized protocol. In case of multiple HBPM within 1 period, the mean systolic and diastolic BP was calculated by dividing the sum of all measurements by the number of BP measurements within that period. At final follow-up, patients were classified as complete, partial or absent clinical/biochemical success using the PASO consensus criteria.^5^ Complete clinical success was defined as HBPM <135/<85 mm Hg without the use of AHT.^13^

### Statistical Analyses

Data were presented as mean±SD for variables that followed a normal distribution and as median (interquartile range) for variables that were not normally distributed. Categorical variables were presented as counts (percentage). Since repetitive follow-up data per patient were collected, a linear mixed-effects model was developed. Follow-up time was entered as fixed effect and the patients were random effects. The Akaike Information Criterion was used for model fitting, since a lower Akaike Information Criterion value indicates better quality of fit. The autoregressive(1) was used as covariance structure, which has homogenous variances and correlations that decline exponentially with distance. This means that the variability in a measurement is constant at every follow-up period, but that 2 consecutive measurements are more correlated than ones further away. The model was adjusted for family history of hypertension, target organ damage, preoperative AVS performed, and the plasma aldosterone concentration. In case a preoperative HBPM was missing, the preoperative daytime ABPM was used as preoperative baseline HBPM value. Patients with at least 2 HBPM or DDD were eligible for the mixed-effects model. Fixed effects estimates were reported with SE and 95% confidence intervals (CI). Statistical analyses were performed with the IBM SPSS Statistics 26.0 software (IBM Corp, Armonk, NY). Figures were generated with GraphPad Prism version 8.3 (GraphPad Software Inc., San Diego, CA).

## RESULTS

### Baseline Characteristics

The prospective database of all consecutive patients with difficult-to-control-hypertension contained 1784 patients. A total of 1156 patients underwent the “Analysis of Complicated Hypertension” diagnostic protocol. Of these 1156 patients, 43 (3.7%) underwent unilateral adrenalectomy for PA. Two of these patients underwent surgery elsewhere and were therefore excluded from the cohort. Baseline characteristics of the 41 included patients are reported in Table 1. Individual patient characteristics are shown in Supplemental Table 1, Supplemental Digital Content 1, http://links.lww.com/SLA/E108. A preoperative HBPM and ABPM with medication was available in 15 patients (mean 158/98 mm Hg) and 26 patients (mean 150/91 mm Hg), respectively. The median preoperative AHT use was 4.2DDD (2.4–6.1). In 39 (95.1%) of the patients the ARR was measured. Thirty-nine (95.1%) of the patients underwent a SIT, which had a positive result for PA in 32 (82.1%) patients and an intermediary result in 7 (17.9%) cases. CT and AVS were performed in 87.8% and 68.3% of the patients, respectively. Twenty-three (56.1%) patients underwent both CT and AVS. All patients underwent a total unilateral adrenalectomy and did not have previous adrenal surgery on the contralateral adrenal gland in their medical history. There were no cases of postoperative adrenal insufficiency (Addison’s disease).

**TABLE 1:** Baseline Characteristics of the Total Cohort

Variable	No.	Values
Age	41	56.2±8.0
Female	41	12 (29.3)
BMI (kg/m^2^)	41	28.6±4.2
Family history of		
Hypertension 1st degree relative <60 y	39	25 (60.1)
Other cardiovascular diseases 1st degree relative <60 y	41	14 (34.1)
HBPM with medication (mmHg)	15	
Systolic		158.2±13.0
Diastolic		97.5±7.9
OBPM with medication (mm Hg)	41	
Systolic		173.6±24.9
Diastolic		101.1±12.2
ABPM with medication (mm Hg)	26	
Systolic		150.2±17.6
Diastolic		90.5±12.9
Systolic daytime		153.7±17.2
Diastolic daytime		92.9±13.0
OBPM without medication (mm Hg)	40	
Systolic		180.0±21.6
Diastolic		106.0±11.1
ABPM without medication (mm Hg)	38	
Systolic		164.5±14.4
Diastolic		99.9±9.2
Need for escape medication	38	10 (26.3%)
Antihypertensive drug use (DDD)	41	4.2 [2.4–6.1]
Number of classes of antihypertensives	41	3.0 [2.0–4.0]
Plasma potassium (mmol/L)	41	3.5 [3.1–3.9]
Potassium supplementation		31 (75.6)
Normokalaemia (≥3.8)		11 (26.8)
eGFR (mL/min/1.73m^2^)	41	80.5 [>60–>90]
Impaired kidney function (<60)		2 (7.7)
Aldosterone-renin-ratio	39	12.5 [7.6–26.8]
Plasma aldosterone concentration (pmol/L)		1210 [690–1530]
Plasma renin activity (fmol/L/s)		66.0 [<40–120]
Saline infusion test	39	
Plasma aldosterone concentration (pmol/L)	39	400 [290–780]
Plasma renin activity (fmol/L/s)	22	46 [<40–63]
CT performed	41	36 (87.8)
Lateralization left		21 (58.3)
Lateralization right		6 (16.7)
Bilateral		3 (8.3)
No lateralization		6 (16.7)
AVS performed	41	28 (68.3)
Lateralization left		15 (53.6)
Lateralization right		12 (42.9)
No lateralization		1 (3.6)
CT+AVS performed	41	23 (56.1)
OSAS	35	12 (34.3)

The number of patients available for analysis (No.) are shown. Values are given as mean (±SD), medians [interquartile range] or counts (percentage).

### Antihypertensive Drug Use in Defined Daily Dose

**FIGURE 1:**
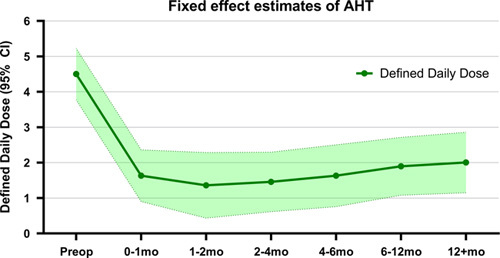
The trend of antihypertensive drug use in Defined Daily Dose from preoperative (baseline) to >12 months postoperative according to the mixed-effects model. Values are reported as means with 95% confidence intervals.

Forty-one patients were eligible for the mixed-effects model analysis. Twenty patients did not need any AHT directly postoperative, of which in 11 patients small dosages of AHT were started during follow-up. Figure 1 and Table 2 show the postoperative trend in AHT use (DDD) estimated using the mixed-effects model. The major decrease in AHT use was seen between preoperative and the first month postoperative measurement – median 4.5DDD (95% CI: 3.8–5.2) to 1.6DDD (95% CI: 0.9–2.4), respectively. After the first postoperative month the median estimated AHT use slightly decreased to 1.4DDD (95% CI: 0.4–2.3) at 1 to 2 months, which was followed by a modest linear increase to 2.0DDD (95% CI: 1.1–2.9) at final follow-up. All postoperative DDD measurements were statistically significantly lower than the preoperative measurement. No statistically significant difference was observed between the various postoperative periods as the CIs overlapped.

**TABLE 2:** Fixed Effect Estimates of Defined Daily Dose and Home Blood Pressure Measurements According to the Mixed-Effects Model

	DDD Estimates		HBPM Estimates			
Period	DDD (SE)	95% CI	SBP (SE)	95% CI	DBP (SE)	95% CI
Preoperative	4.5 (0.3)	3.8–5.2	153 (2.8)	147–158	92 (1.7)	89–96
0–1 mo	1.6 (0.3)	0.9–2.4	140 (3.2)	134–147	85 (2.1)	81–90
1–2 mo	1.4 (0.5)	0.4–2.3	140 (3.6)	133–148	85 (2.5)	80–90
2–4 mo	1.5 (0.4)	0.6–2.3	139 (3.1)	132–145	86 (2.1)	82–90
4–6 mo	1.6 (0.4)	0.8–2.5	136 (3.4)	129–143	86 (2.3)	81–90
6–12 mo	1.9 (0.4)	1.1–2.7	135 (3.1)	128–141	83 (2.0)	79–87
12+ mo	2.0 (0.4)	1.1–2.9	136 (3.6)	129–144	83 (2.5)	78–88

The fixed effect estimates are reported in means.

DBP indicates diastolic blood pressure; SBP, systolic blood pressure.

### Home Blood Pressure Measurements

**FIGURE 2:**
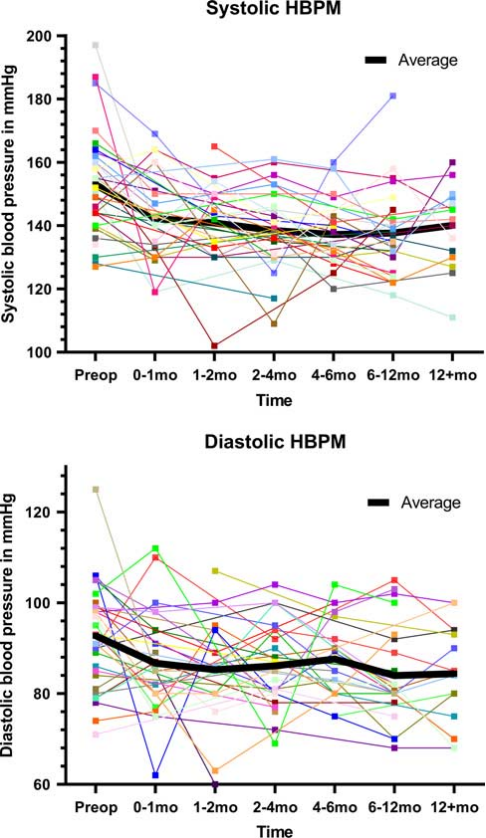
Spaghetti plots: captured systolic and diastolic home blood pressure measurements over time.

**FIGURE 3:**
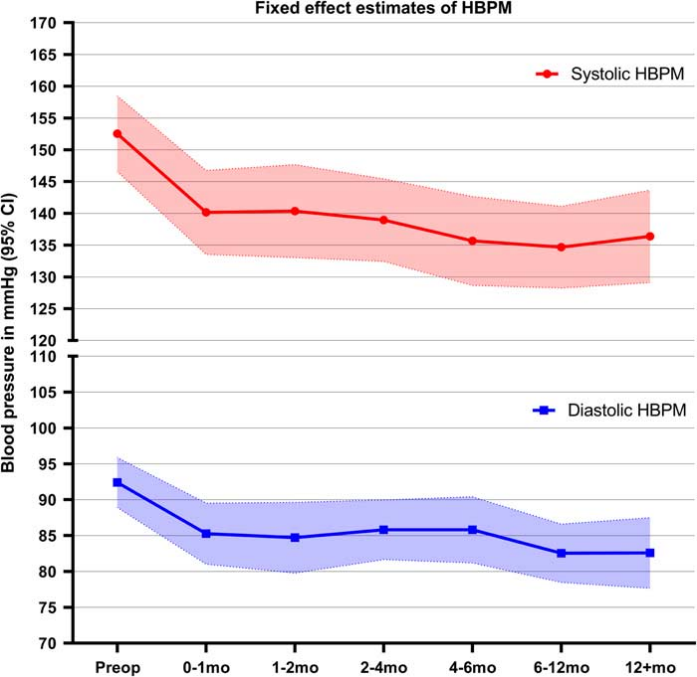
The trend of home blood pressure measurements from preoperative (baseline) to >12 months postoperatively according to the mixed-effects model. Values are reported as means with 95% confidence intervals.

Thirty-six patients were eligible for the mixed-effects model analysis. The mean HBPM for each patient at the respective follow-up intervals was computed from a median of 2 (1–3) measurements. Figure 2 shows HBPM in the year following adrenalectomy. Figure 3 and Table 2 show the postoperative trend in HBPM estimated using the mixed-effects model. Mean estimated HBPM in the first postoperative month was 140/85 mm Hg (95% CI: 134–147/81–90), which considered a mean change of −13/−7 mm Hg compared to preoperative BP. Thereafter, estimated HBPM showed only minor changes in mean systolic and diastolic BP (≤3 mm Hg). All postoperative HBPM were statistically significantly lower than the preoperative measurement. No statistically significant difference was observed between the various postoperative periods as the CIs overlapped.

### Cure Rates

Cure rates could be calculated for 37 patients. Clinical cure, partial clinical success and absent clinical success rates according to the PASO consensus criteria at final follow-up were 4 (10.8%), 29 (78.4%), and 4 (10.8%). In 17 (41.5%) patients, data was available on the postoperative biochemical situation. Of these patients, 16 (94.1%) had complete biochemical success and 1 (5.9%) showed absent biochemical success.

## DISCUSSION

In this longitudinal retrospective cohort study, the postoperative trend in BP-related outcomes (HBPM and AHT) during the first year after adrenalectomy for PA was investigated using a mixed-effects model to account for repetitive measurements, follow-up variability and intermediate missing data.

The trend of AHT use – expressed as DDD – showed that most AHT were successfully discontinued directly after surgery. Twenty patients had no AHT postoperatively, of which in 11 patients eventually small dosages of AHT were restarted during follow-up. In the other patients none or only minor adjustments in AHT were necessary. This resulted in a modest increase in DDD from the second postoperative month until >12 months. Nevertheless, on group level AHT use showed statistically a stable course during the year following adrenalectomy.

HBPM was used to investigate the postoperative BP trend. This trend showed the greatest decrease in BP within the first month after adrenalectomy. Thereafter, HBPM remained stable during the first year of follow-up with only minor fluctuations in mean BP, which resemble the expected BP variability between 2 measurement moments.

The PASO study group and the 2016 Endocrine Society CPG both suggest follow-up up to 1 year after surgery. These suggestions were mainly based on expert opinion.^5^,^7^ This emphasized the need for the current study. The present study provides detailed insight into the trend of AHT use and HBPM at multiple time points during the first postoperative year. The major effect of adrenalectomy on AHT usage and HBPM is achieved short after the operation. This confirms the Endocrine Society CPG expectations stating that BP typically normalizes or shows maximum improvement in 1 to 6 months postsurgery. Furthermore, both AHT and HBPM remained stable during the first year of follow-up with only minor fluctuations. Based on these trends, 1 might suggest that the recommendations for outcome assessment in the PASO study can be brought forward, for example, at 1 month the initial outcome assessment and between 4 and 6 months postoperatively the final outcome assessment. In this way, patients with much improvement in BP, and/or stable BP between initial and final outcome assessment and/or who hardly need any adjustments to their AHT, could be discharged from follow-up more quickly and be further monitored by the family physician. Nevertheless, patients with postoperative persistent difficult-to-control hypertension or rare cases of malignant aldosterone-secreting tumors should receive longer follow-up. Most importantly, this study will aid in patient counseling early after adrenalectomy in their expectations for the months to follow.

The major strength of this study is the prospective routine collection of diagnostic work-up variables, which largely reflect current clinical practice and are the same for all patients. Moreover, most of the patients in this study were diagnosed according to the Endocrine Society CPG and underwent SIT regardless of the ARR outcome, which strongly reduced the influence of verification bias. Another strength is the use of HBPM. Specialists in the field of BP monitoring use the HBPM often in daily clinical practice and treatment decisions are frequently based on the HBPM instead of OBPM. Out-of-office BP measurements (ABPM/HBPM) have the advantage of substantially reducing the white-coat effect and are better in predicting cardiovascular morbidity and mortality. HBPM is more suitable to assess day-to-day BP variability for longer periods of time and to increase patient’s engagement and their adherence to treatment.^13^–^15^ A limitation of the HBPM in this study is that these were not assessed via a standardized protocol, although the mean HBPM was a median of 2 measurements, which should be an accurate representation of the true BP. Therefore, we believe that these HBPM provide a more accurate representation of BP than OBPM. In daily clinical practice in our center, HBPM are used to determine adjustments needed in AHT, both at outpatient and telephone appointments. In addition, e-Health solutions may contribute in validated and protocolized HBPM in the near future, which brings many advantages amongst which the use of telephone consultations and decrease in healthcare costs. A limitation of this study is the relatively low number of included patients. This reflects both the rarity of the disease – as shown in this study with only 3.7% of 1156 patients with difficult-to-control hypertension underwent adrenalectomy – as well as the underdiagnosis of PA in current clinical practice.^16^ Likewise, the potential underrepresentation of the awareness regarding a surgical solution to hypertension by directly initiating treatment with lifelong aldosterone antagonists. This limited sample size may cause insufficient statistical power, nevertheless, on group level no major clinically relevant changes were seen. Furthermore, postoperative HBPM data were stratified into 6 different timeslots of follow-up with available data points ranging from 15 to 25 patients of the total cohort. To overcome this limitation of missing data, we used a linear mixed-effects model which is more flexible in handling intermediate missing data and handling the correlation between repeated measures compared to traditional statistical approaches.^17^,^18^ Another limitation is the low rate of postoperative biochemical evaluation, which was not common practice yet in our center during the entire study period. A general and known limitation is the variability of BP measurements. A random variability in BP measurement (intraobserver and intraindividual) may cause a significant chance of variation when trying to assess whether a treatment is effectively reducing BP.^19^ Intraindividual coefficient of variation for BP measurement were estimated on 9.2% either for systolic and for diastolic BP.^20^ This variability makes it challenging to monitor the postoperative BP, therefore the amount of AHT in DDD was additionally used to assess BP-related outcomes.

## CONCLUSIONS

This study showed that AHT use and HBPM decreased substantially within the first month after adrenalectomy for PA and afterwards generally remained stable during the year following adrenalectomy. This confirms the treatment effect expectations in the Endocrine Society CPG. In addition, we propose that BP-related outcomes can be assessed reliably early after adrenalectomy and question the need for routine long-term follow-up in referral centers in well-regulated patients as these patients could be monitored by their family physician.

## DISCUSSANT

**François Pattou (Lille, France)**

First, I would like to thank the selection committee for the privilege of reviewing this very interesting paper. The authors should be commended for having conducted this precise retrospective analysis of postoperative outcome of adrenalectomy for primary aldosteronism in a single center series of 37 patients. Their data surely confirm the excellent results of surgery resulting in complete biochemical cure in more than 90% of cases and clinical success, according to the current definition, also in 90% of cases. Importantly, the authors showed that successful blood pressure-related outcomes can be reliably assessed as early as one month after adrenalectomy, and later, monitored by primary care physicians. This, therefore, questions the necessity of long follow-up in (expensive) expert referral centers. This is important and very good news for operated patients.

I thank the authors for having carefully addressed most of the comments raised during the reviewing process by the three reviewers. However, I still have a few remaining questions:

First, it is likely that some patients with documented primary aldosteronism from this large cohort of patients with severe hypertension were followed medically and not operated. How different were the baseline characteristics of these non-operated patients and/or the long-term outcomes from those among the operated cases?

Second, ten patients (i.e., 25%) had a final diagnosis of adrenal hyperplasia, alone or associated with an authentical adenoma. Were their blood pressure related outcomes different from those with a single adenoma, especially in the long-term?

Finally, distinguishing a unilateral aldosterone producing adenoma from bilateral hyperplasia is essential. For that purpose, adrenal venous sampling was performed in two thirds of the cases in your study. In order to reduce the need for this invasive procedure, other authors propose Nor-iodo-cholesterol (NP-59) adrenal scintigraphy as an alternative test to confirm unilateral aldosterone production. Do you have any experience with this alternative test? Overall, what strategy do you advise in terms of a lateralization test beyond a CT scan?

**Response From Diederik Suurd (Utrecht, The Netherlands)**

Thank you for your kind comments. To address the first question about the non-operative primary aldosteronism patients, we chose to only focus on the surgically treated patients in this cohort. However, indeed, the other patients in this large database were treated medically. We identified another 23 primary aldosteronism patients, who were all treated medically, in this cohort. This number is not what you would expect to obtain for medically treated patients. You would expect 80 or 90 medically treated patients, but these were not identified in this prospective database. However, the baseline characteristics of these 23 patients did not differ, in terms of preoperative blood pressure and antihypertensives. As I already said, we wanted to determine the effect of surgery. Therefore, we did not extract the long-term follow-up for the medically treated patients.

Second, regarding the different outcomes between adrenal hyperplasia or single adenoma, we didn’t perform a subgroup analysis between those two groups, but we did look at long-term blood-pressure outcomes. The group of patients with adrenal hyperplasia performed similarly to the group with single adenoma. 100% of the patients with adrenal hyperplasia clearly benefitted from surgery.

Third, regarding lateralization techniques, distinguishing between left- or right-sided primary aldosteronism is very important. At our institution, we strive to perform a CT and adrenal venous sampling for every patient. However, for the adrenal venous sampling, we have to refer to another academic hospital in the Netherlands. Sometimes, this is just not feasible. Regarding the NP-59, we have no experience with this technique at our center. That said, currently, we are working on a new PET-CT tracer, named Pentixafor, to see whether this tracer is suitable for distinguishing between left- and right-sided primary aldosteronism. We hope to present the results within the next few months or year.

## Supplementary Material

SUPPLEMENTARY MATERIAL
