# Supplementary material for: Assessing Outcomes After Adrenalectomy for Primary Aldosteronism – Early is Accurate: Retrospective Cohort Study
Source: Ann Surg. 2022 Jul 27;276(5):929–34. doi: 10.1097/SLA.0000000000005639 (PMC9534052; doi:10.1097/SLA.0000000000005639)
Supplement: SUPPLEMENTARY MATERIAL [file sla-276-0929-s001.docx]

**Supplemental Data 1**

*Diagnostic work-up and adrenalectomy for primary aldosteronism*

PA was diagnosed in accordance with the Endocrine Society CPG ^7^. Patients underwent measurement of the ARR and a SIT was performed (regardless of ARR outcome). Plasma aldosterone concentration (PAC; pmol/L) and plasma renin activity (PRA; fmol/L/s) were measured after stopping interfering AHT: at least 6 weeks prior to testing cessation of mineralocorticoid receptor antagonists, and at least 4 weeks prior to testing cessation of diuretics, β-blockers (two-week tapering scheme) and central acting agents (two-week tapering scheme). All other AHT were stopped at least 2 weeks prior to testing and patients were advised to stop non steroid anti-inflammatory drugs and oral contraceptives. During the weeks without AHT, diltiazem and/or doxazosin could be used as rescue medication.

The PAC and PRA were measured in early mornings (0800h – 0900h) in seated position after patients had been up for at least 90 minutes. Afterwards, 2L isotonic saline was infused in 4h and another PAC and PRA measurement was performed in recumbent position. Potassium <3.8 mmol/L was corrected before SIT. The ARR was calculated using the PAC and PRA measurement before SIT. The SIT was positive if PAC was >277 pmol/L, intermediate if PAC was 140-277 pmol/L and negative if PAC was <140 pmol/L.

**Supplemental Table 1**

| **Number** | **Sex** | **Age** | **BMI** | **K^+^** | **ARR** | **PAC** | **PRA** | **CT** | **AVS** | **Surgery, side** | **PA** | **Tumor Size** |
| --- | --- | --- | --- | --- | --- | --- | --- | --- | --- | --- | --- | --- |
| 1 | M | 56 | 27.5 | 4.1 | 8.1 | 310 | 99 | Right | Right | EPRA, Right | APA | 8 |
| 2 | M | 51 | 26.5 | 3.6 | 7.4 | 300 | 62 | N/P | Right | EPRA, Right | NAH | 8 / 6 |
| 3 | M | 63 | 28.6 | 3.9 | 12.3 | 320 | - | Left | No lat. | EPRA, Left | APA/NAH | 8 / 2 |
| 4 | M | 49 | 36.2 | 2.5 | 53.8 | 1560 | <40 | N/P | Left | EPRA, Left | NAH | 15 |
| 5 | F | 36 | 25.0 | 2.9 | 7.1 | 470 | - | Right | N/P | EPRA, Right | APA | 12 |
| 6 | M | 41 | 30.0 | 3.5 | 9.2 | 280 | - | No lat. | N/P | EPRA, Left | PAH | - |
| 7 | M | 54 | 30.0 | 3.7 | 8.4 | 780 | - | Left | N/P | EPRA*, Left | NAH | max. 6 |
| 8 | M | 56 | 35.2 | 3.1 | 7.6 | 630 | - | N/P | Left | EPRA, Left | NAH | 5 |
| 9 | M | 65 | 25.7 | 4.0 | 11.3 | 290 | - | Left | Left | EPRA, Left | NAH | - |
| 10 | M | 52 | 26.3 | 3.1 | 14.7 | 430 | - | Left | Left | EPRA, Left | APA | 11 |
| 11 | F | 73 | 27.0 | 3.3 | 3.0 | 280 | - | Left | N/P | EPRA, Left | APA | 11 |
| 12 | M | 54 | 30.9 | 2.8 | 39.5 | 900 | - | No lat. | Right | EPRA, Right | APA | 12 |
| 13 | M | 63 | 27.5 | 3.9 | 11.3 | 340 | - | Left | Left | EPRA, Left | APA | 14 |
| 14 | F | 48 | 28.4 | 3.0 | 19.8 | 870 | - | Left | N/P | EPRA, Left | APA/NAH | 10 / 10 / 5 |
| 15 | F | 70 | 30.5 | 3.1 | 39.8 | 360 | <40 | N/P | Left | EPRA, Left | APA | 12 |
| 16 | M | 56 | 23.0 | 3.7 | 36.3 | 400 | 47 | Left | Left | EPRA, Left | APA | 8 |
| 17 | M | 48 | 32.6 | 4.4 | 2.8 | 640 | 180 | No lat. | Right | EPRA, Right | APA | 5 |
| 18 | M | 55 | 27.5 | 3.4 | 29.8 | 1180 | <40 | Left | Left | EPRA, Left | APA | 12 |
| 19 | M | 52 | 26.3 | 3.8 | 26.8 | 500 | <40 | Right | Right | EPRA, Right | APA | 11 |
| 20 | M | 59 | 29.1 | 3.9 | 36.1 | 1350 | <40 | Left | Left | EPRA, Left | APA | 11 |
| 21 | M | 61 | 27.8 | 4.2 | 14.8 | 320 | <40 | Left | Left | EPRA, Left | NAH | 6 |
| 22 | F | 57 | 31.2 | 3.3 | 46.0 | 560 | <40 | No lat. | Right | EPRA, Right | APA | 10 |
| 23 | F | 49 | 25.3 | 3.5 | 17.6 | 1860 | 55 | N/P | Left | EPRA, Left | APA | 9 |
| 24 | M | 58 | 22.6 | 3.7 | 28.3 | 570 | 57 | Left | Left | EPRA, Left | APA | 10 |
| 25 | M | 56 | 27.7 | 3.5 | 26.4 | 370 | <40 | Right | Right | Open**, Right | APA | 4 |
| 26 | F | 52 | 24.0 | 3.4 | 24.8 | 520 | <40 | Right | Right | EPRA, Right | APA | 9 |
| 27 | V | 51 | 27.1 | 3.0 | - | - | - | Left | N/P | EPRA, Left | APA | 4 |
| 28 | M | 54 | 24.5 | 3.9 | - | - | - | Left | N/P | EPRA, Left | APA | 13 |
| 29 | M | 56 | 30.6 | 3.5 | 11.3 | 420 | - | Left | N/P | EPRA, Left | APA | 10 |
| 30 | M | 57 | 28.9 | 3.0 | 25.8 | 220 | <40 | Both | Left | EPRA, Left | APA | 10 |
| 31 | M | 53 | 40.3 | 3.0 | 6.9 | 1140 | 60 | Left | Left | EPRA, Left | APA | 10 |
| 32 | V | 69 | 20.8 | 3.9 | 10.3 | 1030 | 52 | Left | Left | EPRA, Left | APA | 16 |
| 33 | M | 48 | 24.4 | 3.9 | 4.2 | 270 | - | Left | N/P | EPRA, Left | APA | 10 |
| 34 | M | 62 | 34.7 | 3.2 | 12.5 | 170 | - | Both | Right | EPRA, Right | APA/NAH | 10 / 7 |
| 35 | M | 69 | 26.0 | 3.3 | 16.7 | 300 | 73 | Both | Right | EPRA, Right | NAH | - |
| 36 | V | 43 | 39.3 | 3.4 | 6.3 | 320 | 46 | No lat. | Right | EPRA, Right | APA | 11 |
| 37 | M | 58 | 32.7 | 3.5 | 35.0 | 210 | - | Left | N/P | EPRA, Left | NAH | - |
| 38 | V | 72 | 27.1 | 3.4 | 10.6 | 270 | - | Left | N/P | EPRA, Left | APA/NAH | 9 |
| 39 | V | 57 | 29.0 | 3.7 | 6.7 | 240 | - | Left | N/P | EPRA, Left | NAH | - |
| 40 | M | 62 | 28.8 | 3.4 | 5.1 | 180 | 110 | Right | N/P | EPRA, Right | APA | 16 |
| 41 | M | 58 | 25.5 | 3.4 | 13.5 | 1110 | 67 | No lat. | Right | EPRA, Right | APA | 8 |

***Table 2 - Overview of individual baseline characteristics*** ** = converted to open surgery because of lack of overview
** = previous ipsilateral partial adrenal laparoscopic surgery
PAC and PRA values are measured after the saline infusion test. Potassium values were measured without stopping supplementation. Tumor size in millimeters.
Abbreviations: BMI, body mass index in Kg/m^2^; K^+^, potassium in mmol/L; ARR, aldosterone-to-renin ratio; PAC, plasma aldosterone concentration in pmol/L; PRA, plasma renin activity in fmol/L/s; CT, computed tomography; AVS, adrenal venous sampling; PA, pathology; M, male; F, female; EPRA, endoscopic posterior retroperitoneal adrenalectomy; APA, aldosterone-producing-adenoma; NAH, nodular adrenal hyperplasia; PAH, primary adrenal hyperplasia; N/P, not performed; No lat., no lateralization.*
